# Identifying Determinants of Dyslexia: An Ultimate Attempt Using Machine Learning

**DOI:** 10.3389/fpsyg.2022.869352

**Published:** 2022-04-07

**Authors:** Sietske Walda, Fred Hasselman, Anna Bosman

**Affiliations:** Behavioural Science Institute, Radboud University, Nijmegen, Netherlands

**Keywords:** dyslexia, cognitive skills, reading development, machine learning, word decoding and reading outcomes

## Abstract

Research based on traditional linear techniques has yet not been able to clearly identify the role of cognitive skills in reading problems, presumably because the process of reading and the factors that are associated with reading reside within a system of multiple interacting and moderating factors that cannot be captured within traditional statistical models. If cognitive skills are indeed indicative of reading problems, the relatively new nonlinear techniques of machine learning should make better predictions. The aim of the present study was to investigate whether cognitive factors play any role in reading skill, questioning (1) the extent to what cognitive skills are indicative of present reading level, and (2) the extent to what cognitive skills are indicative of future reading progress. In three studies with varying groups of participants (average school-aged and poor readers), the results of four supervised machine learning techniques were compared to the traditional General Linear Models technique. Results of all models appeared to be comparable, producing poor to acceptable results, which are however inadequate for making a thorough prediction of reading development. Assumably, cognitive skills are not predictive of reading problems, although they do correlate with one another. This insight has consequences for scientific theories of reading development, as well as for the prevention and remediation of reading difficulties.

## Introduction

Dyslexia is characterized by severe problems with learning basic subskills of reading and spelling, often resulting in delays in broader literacy skills and unequal opportunities in education and society. In the Netherlands, approximately 10–15% of children in primary and secondary education are diagnosed with dyslexia (Inspectie van het Onderwijs, 2019). Research indicates that dyslexia is sometimes associated with impairments in various cognitive skills, such as phonological awareness, orthographical awareness, syntactic awareness, working memory, as well as general cognitive deficits such as impairments of attention, rapid naming, and self-control (Vellutino et al., 2004; Catts, 2017).

The role of cognitive skills in dyslexia has been subject to varying interpretations. Some authors advocate the existence of different types of reading problems, explaining them by a dysfunction of different cognitive functions. Others interpret these cognitive dysfunctions as a consequence of a more general deficit that causes both cognitive dysfunctions and reading problems (Parrila et al., 2019). Most theories, however, suggest that dyslexia is caused by some sort of brain dysfunction, resulting in an “all or nothing” diagnosis of dyslexia: Children with the brain dysfunction do and children without the brain dysfunction do not have dyslexia. Still other theories suggest that symptoms of dyslexia result from a multi-factorial interplay of (genes), cognitive skills and environment, resulting in a continuum of more to less severe reading and spelling problems (Vellutino et al., 2004; Catts, 2017).

The relevance of the role of cognitive skills in reading problems differs between varying perspectives. From a theoretical perspective, knowledge about cognitive skills involved in learning to read can lead to insights in the nature of reading and processes that accompany the development of automated reading skill (e.g., see Hammill, 2004). From a prevention perspective, knowledge about cognitive skills predictive of reading difficulties can help identify children at risk for developing reading difficulties. If children at risk are identified early, instruction and exercises can be adjusted to lower the chance of developing reading problems (e.g., see Hammill, 2004; Al Otaiba and Fuchs, 2006; Stuebing et al., 2015). From a remediation perspective, it is argued that knowledge about cognitive skills affecting the development of automated reading skill will provide more effective interventions for children lagging behind. When strengths and weaknesses on these particular cognitive skills are known, they could be part of the remediation process. This knowledge is of great importance, because a substantial part of children with dyslexia receiving early literacy intervention respond below expectations and are labeled treatment resisters (e.g., see Al Otaiba and Fuchs, 2006; Stuebing et al., 2015).

The aim of the present study was to find cognitive factors of varying origin that play a role in the development of reading skill in children with dyslexia and children with typical reading development. In this introduction, we will first discuss results of previous research (see Table 1). Previous research demonstrates two concerns: (1) empirical identification of causal factors, and (2) the application of research models adequate for identifying factors involved in reading *level* and in reading *progress*. Next, ideas on potential models of reading development are considered, resulting in a discussion of potential utility of machine learning techniques in an ultimate attempt to identify cognitive skills that are involved in learning to read. Finally, we will present the aims and research questions of the present study.

**TABLE 1:** Results of meta-analyses on the relation between reading level and cognitive skills, and reading progress and cognitive skills.

Article	Measure	Study aim	Sample characteristics*^a^*	Cognitive skill *^b^*	*k*	*Effect Size^c^* ^,*d*^	*I*^2^ *^e,f^*
Hammill and McNutt, 1981	Reading level and progress	Concurrent and longitudinal (after > 1 year) correlation with word recognition, composite reading or reading comprehension*^g^*	Predominantly mixed samples; few learning disabled/disabled reader; aged Kindergarten 2, Grade 3-12, and other. Language: n.s.; Orthography: n.s.	General academic achievement Phonics knowledge Intelligence Readiness Spoken Language Perceptive abilities Motor generalizations Affect (emotional functioning)	14 / 14 4 / n.s. 100 / 25 20 / 19 8 / 3 14 / 4 19 / 5 30 / 8	0.85 / 0.74 0.71 / n.s. 0.61 / 0.52 0.56 / 0.50 0.51 / 0.48 n.s. / n.s. n.s. / n.s. n.s. /.25	n.s. n.s. n.s. n.s. n.s. n.s. n.s. n.s.
Swanson et al., 2003	Reading level	Concurrent correlation with word reading (real and pseudowords)	Average and poor readers; Language: English, Dutch Orthography: n.s.	Spelling Pseudoword reading Phonological awareness Rapid naming Vocabulary Orthography IQ Memory span	6 24 194 107 37 61 35 46	0.78 0.69 0.43 0.42 0.38 0.41 0.42 0.37	0.00 79.02 74.03 68.46 58.16 72.23 55.99 25.26
Melby-Lervåg, 2012	Reading level	Concurrent correlation with text decoding	Average school-aged; Language: n.s.; Orthography: n.s.	Phoneme awareness Rhyme awareness Verbal short-term memory	7 7 7	0.56 0.41 0.28	67.64 67.01 51.21
Parrila et al., 2019	Reading level	Comparison of children with and without dyslexia (reading level-matched [RL]and chronological age-matched [CA])*^h,i^*	Children with dyslexia and reading level controls (<13 years) Language: Finnish, Greek, Spanish, Hungarian, Icelandic, Italian, Swedish, Norwegian, German; Orthography: highly consistent European alphabetic	Phonological awareness Nonword reading Rapid naming Verbal short term memory Auditory temporal processing	19 / 19 12 / 10 14 / 14 12 / 12 5 / 5	–0.21 /–0.49 –0.05/–0.76 –0.01 / –0.57 0.09 / –0.58 –0.24 / –0.21	87.31 / 83.52 89.71/91.94 74.61 / 80.43 85.72 / 86.69 85.56 / 76.88
Parrila et al., 2019	Reading level	Comparison of children with and without dyslexia*^j^*	Dyslexic readers; age-matched controls; (age 7–37) Language: English, French, Italian, Finnish, Spanish, Dutch, German, European-Portuguese, Chinese Orthography: “opaque,” “transparent,” “medium”	Rapid automatized naming: - accuracy - fluency - letters - numbers - objects - colors	21 216 67 127 93 56	0.23 0.50 0.50 0.54 0.54 0.32	9.91 78.53 86.81 83.24 75.12 64.39
Araújo and Faísca, 2019	Reading level	Comparison of children with and without dyslexia*^i^*	Children with and without dyslexia; (age 5–18) Language: Dutch, English, German, Norwegian, Italian, Greek, French, Brazilian, Portuguese, Polish, Algerian, Chinese Orthography: n.s.	Inhibition - reaction time - error rate - accuracy Switching attention - reaction time - error rate - accuracy Auditory working memory - accuracy	10 5 2 3 6 2 14	0.29 0.24 0.48 0.33 0.41 0.72 0.48	0.00 0.00 0.00 0.00 0.00 0.00 0.00
Scarborough, 1998	Reading progress	Correlation with future (after 1, 2, or 3 years of instruction) word reading, composite reading score or rarely reading comprehension	Unselected samples, few high risk samples; Language: n.s.; Orthography: n.s.	Concepts of print Letter-sound and reading skills Letter identification Phonological awareness Speech discrimination Speech production IQ full scale Verbal IQ Performance IQ Receptive vocabulary Expressive vocabulary Rapid naming: - Colors, objects - Digits, letters Receptive language skills - Syntax/morphology - Semantic/unspecified Expressive language skills Verbal memory - Words, digits - Story, sentences Visual perception Visual-motor integration Visual memory (Motor skills)	7 22 24 27 11 4 11 12 8 20 5 9 8 9 11 11 18 11 5 6 8 5	0.46 0.56 0.52 0.46 0.22 n.s. 0.41 0.37 0.26 0.33 0.45 0.37 0.41 ≤0.37 0.24 0.32 0.33 0.45 0.22 0.16 0.31 0.25	0.00 0.00 0.00 0.00 0.00 0.00 0.00 0.00 0.00 0.00 0.00 0.00 n.s. 0.00 0.00 0.00 0.00 0.00 0.00 0.00 0.00
Nelson et al., 2003	Reading progress	Treatment effectiveness of early literacy interventions	Students at risk for reading disabilities; Preschool-3rd Grade; Language: German and n.s. Orthography: n.s.	Rapid naming (Problem behavior) Phonological awareness - phonemic - rhyming Alphabetic principle Memory - short term - long term IQ (Demographic) - (disability / retention) - (ethnicity) - (grade)	7 6 17 13 4 18 11 8 3 8 5 3 1 1	0.47 0.43 0.40 0.35 0.49 0.34 0.30 0.29 0.32 0.25 0.07 0.10 0.10 –0.24	0.00 0.00 0.00 0.00 0.00 0.00 0.00 0.00 0.00 0.00 0.00 0.00 0.00 0.00
Tran et al., 2011	Reading progress	Comparing reading level of responders and low-responders during interventions in reading: pretest and posttest*^k^*	Children at risk for reading disabilities; Language/orthography: n.s. Orthography: n.s.	General IQ Verbal IQ Real-word identification Rapid naming speed Phonological awareness Pseudo word reading Vocabulary Reading comprehension (Spelling) Phonological memory (Behavior) (Reading fluency) (General reading achievement)	2/9 1/3 21/11 13/15 28/9 19/11 4/8 8/18 1/1 2/8 6/6 2/2 1/2	0.36 / 0.11 0.34 / 1.07 1.06 / 1.53 1.31 / 0.74 1.15 / 0.82 1.10 / 1.28 0.71 / 1.19 0.45 / 1.43 1.85 / 0.79 0.41 / 0.92 0.15 / –0.51 0.70 / 0.66 –0.32 / –0.50	0.00 / 91.56 n.s. / 79.25 77.99 / 79.40 82.02 / 0.00 85.25 / 69.21 89.24 / 88.59 0.00 / 93.83 70.56 / 72.56 n.s. / n.s. 0.00 / 64.77 81.82 / 72.97 95.03 / 94.99 100 / 0.00

*n.s. = not specified.*

*^a^ Information about age/Grades, dyslexia diagnosis, and language or orthography (when specified).*

*^b^ Skills that were reported by the articles, but cannot be considered cognitive skills are placed between brackets.*

*^c^ Effect sizes in the meta-analyses were reported using d, g, and r. In order to ease comparison of effect sizes between meta-analyses we inferred r from the information provided by the authors. When d measures were reported as effect size metric, r was inferred using the formula: $r=\frac{d}{\sqrt{d^{2}}+a};$ when g measures were reported as effect size metric, r was inferred using the formula: $r=\frac{g}{\sqrt{g^{2}}+a}$, where a is a correction factor that depends on the ratio of the sample sizes. Tran et al. (2011) provided only d as effect size and provided insufficient information to infer r. Therefore, d measures are displayed in the effect size column for the meta-analysis of Tran et al. (2011).*

*^d^ All meta-analyses provided mean weighted effect sizes, except Hammill and McNutt (1981), who reported median correlation coefficients, only when correlations were significant.*

*^e^ When Q measures were reported as homogeneity metric, I^2^ was inferred using the formula: $I^{2}=\frac{Q-\left(k-1\right)}{Q}\times 100\%$ for Q > (k - 1), I^2^ = 0 for Q ≤ (k – 1), where k is the number of studies. I^2^ is used to quantify heterogeneity among the studies included in a meta-analysis, and is defined as “a percentage of heterogeneity, that is, the part of total variation that is due to between-studies variance τ^2^.” (Huedo-Medina et al., 2006, p. 197). Homogeneity statistics could not be derived for results of Hammill and McNutt (1981), because they reported median correlation coefficients as effect size metric.*

*^f^ Please note that I^2^ is biased when the number of studies is small. Interpretation of I^2^ is problematic when k < 20 see Huedo-Medina et al. (2006).*

*^g^ results on concurrent concurrent reading / results on longitudinal reading.*

*^h^ for comparison with RL group / for comparison with CA group.*

*^i^ positive correlations indicate highest group means for the dyslexia group.*

*^j^ positive correlations indicate highest group means for the (non-dyslexic) control group.*

*^k^ for pretest reading level / for posttest reading level.*

The relationship between reading level and various cognitive skills has been well established by previous research. The statistical technique most frequently used for identifying factors that are important in the process of learning to read is correlation analysis. Even though correlations are bivariate and cannot determine any directional or causal influences, researchers assume that a correlational analysis provides information concerning factors that might be involved in learning to read. In search for causal factors, significant correlations are insufficient to establish causal relationships with learning to read. However, researchers assume that in the search for causal factors, significant correlations should at least be present (corresponding to the fifth axiom of Spinoza, 1678/1928, p. 15). Therefore, factors that prove to correlate with reading level are of interest for researchers who seek to find causal relationships between cognitive factors and reading skill (Hammill, 2004; Stuebing et al., 2015).

In the first three rows of Table 1 meta-analyses on cognitive skills and reading level are presented. Several cognitive skills tend to correlate significantly (not necessarily substantially) with reading level. Age or reading skill often have moderating effects on these correlations (see Hammill and McNutt, 1981; Swanson et al., 2003; Melby-Lervåg, 2012). However, there are three issues that impede the interpretation of correlation between cognitive skills and reading level: (1) For some variables, the amount of overlap with reading skill is unclear. For example, variables such as academic achievement, spelling, pseudoword reading and letter identification could be considered reading skills or at least literacy skills. These skills rather mirror subskills of reading and literacy than function as independent potential cognitive determinants of reading skill. (2) Correlation analyses have not yet led to desirable correlations between cognitive determinants and reading level in such a way that they imply an exhaustive explanatory model of reading skill. Skills close to the reading process (e.g., spelling, pseudoword reading, letter identification) correlate more strongly with reading level than with other cognitive skills (e.g., IQ, memory, attention). Still, correlation coefficients between cognitive skills and reading level rarely exceed *r* = 0.50 (see Table 1), which corresponds to a proportion of explained variance of 0.25; thus 75% of variance remains unexplained. (3) Indices of heterogeneity between studies, when specified and not biased by small samples of studies, indicate a medium to high degree of heterogeneity between studies included in these meta-analyses (see Swanson et al., 2003; Melby-Lervåg, 2012; based on Huedo-Medina et al., 2006). High heterogeneity among the selected studies indicates that fixed effects models, applied when interpreting unique effect sizes, are unsuitable for a comparison of results, because effect sizes tend to differ between studies and as such, results of the studies included in these meta-analyses are not unequivocal.

Several authors studied discrepancies in cognitive skills of children with dyslexia and typically developing children. The meta-analyses displayed in rows 5–7 of Table 1 indicate that children with dyslexia have an increased chance of lower abilities on several cognitive skills compared to typically developing children. Effect sizes (converted to correlation measures in Table 1) suggest that cognitive skills of children with dyslexia tend to differ from cognitive skills in typically developing children, although indices of heterogeneity (when specified and not substantially biased) again indicate that results were not unequivocal (Araújo and Faísca, 2019; Lonergan et al., 2019; Parrila et al., 2019). Meta-analyses that distinguished age-matched controls from reading-matched controls, however, reveal that cognitive skills of children with dyslexia do not significantly differ from those of reading-matched controls (e.g., Araújo and Faísca, 2019; Parrila et al., 2019). This indicates that at least some of these lower abilities might have resulted from lower reading level by origin, that is, implying circular causality. On that note, it is suggested that children with dyslexia are identified by decoding skills (i.e., translating printed words in speech) in itself rather than by cognitive skills.

Thus, several cognitive skills tend to correlate with reading level and children with dyslexia tend to differ from age-matched controls in their cognitive skills. Considering the relationship between cognitive skills and reading level, it can be concluded that the relationships are evident, although the magnitudes of these relationships are limited.

Investigating the relationship between reading *progress* and cognitive skills is less straightforward than the relation with reading level. As discussed in the previous paragraph, correlations between reading level and cognitive skills indicate possible determinants or causal factors of reading progress: In order to be predictive of future reading progress, a factor should at least correlate to some extent with present reading level (e.g., Nelson et al., 2003; Tran et al., 2011). However, even when factors correlate with reading level, one cannot exclude the possibility of the effects of a third variable, influencing both cognitive skills and reading level, and thereby generating a correlation between them. Also, correlations never express directions of relationships between variables: The relationships could be opposite to what was expected (instead of cognitive skills causing variations in reading level, reading level might be causing variations in cognitive skills), or being bidirectional (cognitive skills and reading level might mutually influence each other over time). Bidirectional relationships between variables form a considerable possibility in research on reading skill, as is evidenced by the relationship between word reading and phonological awareness (see Castles and Coltheart, 2004). According to Catts (2017), such bidirectional relationships could inflate the correlations that were found over time. Catts probably meant to point out that a correlation measure is not suitable to indicate the strength of a causal relationship: One obvious reason is that factors with reciprocal relationships will produce strong correlations over time when they keep mutually influencing each other all the time. Thus, correlations between reading level and cognitive skills do not necessarily indicate that (lacking) cognitive skills cause lower reading level. To predict reading development, a factor should at least be associated with gains in reading skill over time. In other words, in order to prove that a factor affects reading development, its causal role should be observed in an experiment. In the field of reading development, possibilities are limited, because participants with dyslexia and typical reading development cannot be randomly assigned to groups. Therefore, only quasi-experimental designs can meet this demand. Several authors discuss possibilities for quasi-experimental designs, resulting in roughly three suggestions, listed by ascending validity in proving causal relationships:

(1) Models that explain variance in growth in reading/spelling over time by cognitive skills at baseline (unconditional models, see Stuebing et al., 2015);
(2) Models that explain outcome of reading/spelling skills by baseline reading/spelling skills as well as baseline cognitive skills (conditional models, see Stuebing et al., 2015);
(3) Models that explain progress in reading/spelling skills from progress in cognitive skills (see Vellutino et al., 2004).

Unconditional models fit theoretical research questions about correlations between baseline cognitive skills and reading/spelling development, but do not aim at explaining any causal relationships. If these correlations prove to be compelling, one could ask whether baseline cognitive skills could add to baseline reading/spelling skills during an intervention, which would fit the conditional model. Vellutino et al. (2004), however, argue that possible causal relationships can only be inferred from a model that explains progress in reading skill from progress in cognitive skills. This would be the only opportunity to approximate the demand of observing the causal trajectory in an (quasi-)experiment.

Research results on the relationship between reading progress and cognitive skills are less common and less clear than research results on the relationship with reading level. Overall, studies using unconditional models tend to identify more baseline cognitive skills related to outcome reading level measures and stronger relationships than studies using conditional models (e.g., Stuebing et al., 2015). Although some studies indicate that a number of baseline cognitive skills are related to outcome reading-level measures, it is questionable to what extent these cognitive skills are truly separable from reading skill itself. Meta-analyses on the relation between baseline cognitive skills and progress in reading are presented in the first row and the last three rows of Table 1. The results of the meta-analyses presented in Table 1 originated from the results on unconditional models, albeit Tran et al. (2011) also presented results on conditional models. These meta-analyses reveal little evidence for a relationship between baseline cognitive skills and progress in reading skill. Factors that were most strongly related to results of early literacy training overlapped with reading skill (e.g., general achievement, word reading, pseudoword reading, and reading comprehension), whereas other cognitive skills (e.g., IQ, memory, rapid naming speed, and phonological awareness) produced less strong results (see Hammill and McNutt, 1981; Scarborough, 1998; Nelson et al., 2003; Tran et al., 2011).

Again, when specified, indices of heterogeneity between studies included in the meta-analyses indicated that results were not unequivocal. Findings from empirical research provide suggestions for causes for the varying results on the relationship between reading (level and progress) and cognitive skills. Studies tend to differ in sample characteristics, measurement instruments, methodological approach, and study design (e.g., see Vellutino et al., 2004; Stuebing et al., 2015). As such, comparing studies on the role of cognitive skills in reading development seems like comparing apples to oranges. Thus, research on the relationship between reading progress and cognitive skills is limited in quantity and methodological strength. Moreover, little evidence is found for a relationship between cognitive skills and progress in reading skill and results tend to vary between studies.

Analyses based on traditional linear techniques such as correlations and variances, as presented in the previous paragraphs, may not be applicable to a multifactorial and multidirectional system such as reading development. As Parrila et al. (2019) state, no single factor alone can be accountable for development of a skill as complex as reading, especially not regarding development over time. Even traditional multi-factorial approaches do not have the capacity to encompass all possible factors and relationships (e.g., Connor and Morrison, 2017). Catts (2017) proposed that dyslexia could be the outcome of multiple interacting factors, moderated by several positive and negative influences. Dyslexia, then, is understood as a condition that follows the risk-resilience framework, in which specific factors serve as moderators of risk, determining different outcomes in individuals with similar precursors (Catts, 2017).

Research based on traditional linear techniques has not been able to clearly identify the role of cognitive skills in reading problems, presumably because the process of reading and the factors that are associated with reading reside within a system of multiple interacting and moderating factors that cannot be captured within traditional statistical models. In contrast, the view of a broad set of interacting variables fits the model of complex adaptive systems: “(…) systems that have a large number of components, (…), that interact and adapt or learn” (Holland, 2006, p. 1). The most important characteristics of complex adaptive systems are:

(1) Components act simultaneously;
(2) Components only act upon rules (specific conditions e.g., actions of other components, environmental circumstances);
(3) Within a component, several rules can combine into specific sequences of rules to, for example deal with novel situations;
(4) Components can adapt over time, that is, they change their actions in order to abide to the rules and the sequences of rules. Usually these changes are not random, but designed to improve the outcome based on prior experiences (Holland, 2006).

If lags in cognitive skills play a critical role in the emergence and persistence of reading problems, analyses based on complex adaptive systems should be able to at least identify which cognitive skills are involved, and possibly also how and to what extent they are involved. A relative novel way of investigating complex systems is the use of machine learning, in which a set of data together with a set of algorithms seek to find the best solution given the data. Machine learning roughly falls apart in two types of learning: unsupervised and supervised. Unsupervised learning is used to find patterns in the input data without using any output data, particularly to find clusters or dimensions. In supervised learning, the model is confronted with input and output data in order to find the best function between them. Supervised learning is mostly used for predicting future events (Russell and Norvig, 2010).

Research applying the technique of machine learning to the field of reading skill is scarce. To our knowledge only two studies have been published, both applying the unsupervised learning technique of Self Organizing Maps (SOM; Loizou and Laouris, 2010; Astle et al., 2018). Loizou and Laouris (2010) used the SOM technique to make different clusters of participants based on measures of cognitive skills and word reading skill. Subsequently, they identified which (sub)tests made the strongest contribution to the assignment of participants to clusters. Their results showed that information of only four tests (auditory memory, navigation, word identification and word attack, and rapid naming of pictures) were sufficient to classify 94.64% of the participants in the identified clusters. Also, the fifth strongest factor contributing to the classification was age. Astle et al. (2018) adopted a slightly different approach. They used the SOM technique to distinguish between different clusters of participants based only on measures of cognitive skills (“cognitive profiles,” based on seven measures: nonverbal reasoning, vocabulary, phonological processing, and four measures of short-term memory) and afterwards compared these clusters to initial referral routes and diagnoses. Their results showed that participants allocated to the cluster “lag in a broad spectrum of cognitive skills,” showed the most severe problems in reading, spelling, and math skills. More importantly, learning-problems characteristics and how participants were diagnosed were not related to the cognitive profiles determined by the SOM technique. Thus, some efforts were made studying the role of cognitive skills in reading skill, revealing some preliminary results: Only a small number of cognitive and reading skills were needed to make up some clusters, albeit when clusters were based on cognitive skills only, they were not related to learning skills.

Although both studies on the role of cognitive skill in reading development did consider the multifactorial nature of reading skill, neither was capable of identifying cognitive factors predictive of reading skill. These studies used the unsupervised learning SOM technique, which aims at classification, and both input (cognitive) variables and output (reading level) variables are from the same moment of measurement. As such, participants are labeled according to their reading level, and not according to their reading progress. To come back to the relevance of the role of cognitive skills in reading problems, this mainly serves the theoretical perspective about the nature of reading and processes that accompany the development of automated reading skill. Questions such as which cognitive skills are predictive of reading difficulties (prevention perspective) and which cognitive factors can affect the development of automated reading skill (remediation perspective) are left unanswered.

To answer these questions, supervised machine learning techniques should be applied, because these techniques are capable of making predictions about future events (Russell and Norvig, 2010; Lantz, 2019). More specifically, machine learning based on neural networks seems the most likely technique to identify cognitive factors predictive of reading skill. According to Lantz (2019), neural networks belong to so-called black-box methods and these are, more than any other technique, capable of modeling complex patterns. Moreover, neural networks pose few assumptions on the input data.

The aim of the present study was to find cognitive factors of varying origin that play a role in the development of reading skill in children with dyslexia and children with typical reading development. The present study will address the following research questions:

1. To what extent are cognitive skills indicative of present decoding level?
2. To what extent are cognitive skills predictive of future decoding progress?

The present study is unique in applying supervised machine learning techniques to the field of reading development. Moreover, the present study includes analyses on a relatively homogeneous group of reading disabled children as well as a heterogenous group representing a sample of the population of school age children to correct for the effects of restriction of range.

## General Methods

### Overview

**FIGURE 1:**
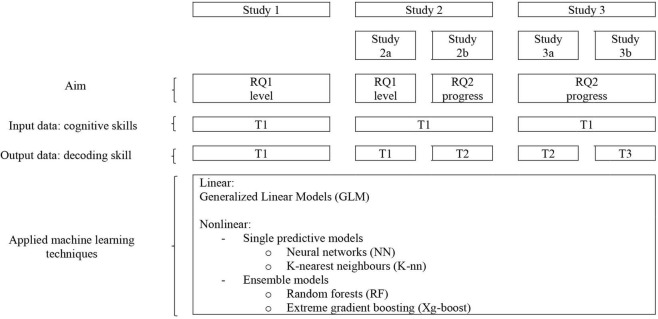
Schematic overview of conducted studies and aims, research questions, and model building techniques that were involved. RQ, research question.

Three datasets will be subjected to machine-learning modeling and reported on in three studies. Study 1 was performed on the dataset of Verhoeven and Keuning (2018) on cognitive precursors of reading and reading level of 2007 Dutch primary-school children. For Study 2, data of a previous study (Walda et al., 2014) were used and supplemented with new cases (i.e., more participants with dyslexia) and new cognitive skills variables, that is, in the Walda et al. study a number of specific executive functions were investigated, whereas the current study 2 considers cognitive skills in a more general way (noem hier wat voorbeelden van specifiek en algemeen). The dataset of the present study contained data on cognitive skills, reading and spelling in 383 Dutch children with dyslexia. The data consist of results on cognitive precursors of reading and reading level prior to reading and spelling remediation as well as reading level after three months of reading and spelling remediation in a Dutch clinic for the assessment and remediation of learning disorders. Study 3 was conducted on the dataset of Braams and Bosman (2000) pertaining to kindergarten predictors of reading and spelling level of two cohorts (117 and 82, respectively) Dutch primary-school children in Grade 1. Study 1 was aimed at answering research question 1. Studies 2 and 3 also aimed at answering research question 1 (Study 2a and Study 3a) as well as research question 2 (Study 2b and Study 3b). For a schematic overview of the particular characteristics of the studies, see Figure 1.

Although the original datasets contained more cases, only results on complete cases are presented in the present study: Cases with a missing record on one or more variables (word decoding or one of the cognitive skills variables described in Supplementary Material B) were excluded from the analyses because they do not contribute to the building of a model. Concerning research question 1 (word decoding level) in Studies 1, 2a, and 3a, input data of cognitive skills on T1 were modeled on output data of word decoding skill at T1. Concerning research question 2 (word decoding progress) in Studies 2b and 3b, input data of cognitive skills on T1 were modeled on output data of word decoding level at T2, corresponding to what was considered an unconditional model in the “Introduction” section.

### Data Analysis

The models were trained in R (CRAN version). The data of Studies 1 and 2 were normalized to a zero to one range. Those of Study 3 had to be normalized using Z-scores to be able to compare between participants. To maximize the opportunity of selecting a model with a good fit, each dataset was subjected to four different techniques of supervised machine learning. Two techniques building single predictive models were used, namely, neural networks and k-nearest neighbors and two ensemble techniques, namely, random forests and extreme gradient boosting (Xg-boost). In addition, a model using General Linear Model (GLM) technique was used to compare the results of all four nonlinear techniques to a linear one. For a description of the Xg-boost technique, see Lesmeister (2019); for a description of the other four techniques see Lantz (2019).

#### Model Building

Each model was built to result in a topology with several input factors measuring cognitive skills, which predicted one outcome measure, that is, word-decoding level. To compare the fit of most models to the data, outcome measures were treated binomially, distinguishing between participants with the 20% lowest word-decoding raw scores and participants with the 80% best word-decoding raw scores in Studies 1 and 3. In Study 2, standardized scores (i.e., c-scores) were used for the outcome measure, resulting in binomial scores distinguishing between c-scores 0 and 1 and c-scores 2–9, which corresponds to approximately 10% lowest word-decoding efficiency and 90% highest scoring in the regular population.

Parameters of the four models were tuned until they approximated the model that best fit the data in the dataset (for more information about building these models see Lantz, 2019; Lesmeister, 2019). Which parameters can be tuned depends on the specific machine learning technique, and is specified in Supplementary Material A. In Studies 1 and 2, 90% of the datasets were used for tuning and training and 10% for testing the model. Because of the relatively small sample of data in Study 3, 75% of the dataset was used for tuning and training and 25% for testing the model. To overcome the problem of differential findings as a result of the seed set, a number of models were built for each technique and results were run on 100 different seeds. Results are reported with a 95% confidence interval. For more details about building, tuning, training, and testing of the models see Supplementary Material A.

#### Model Evaluation

Subsequently, model performance was evaluated computing summary statistics for the predictive ability of the model and visualizing performance tradeoff for all five (one linear and four nonlinear, see Figure 1) models. We used R’s set.seed function (R Core Team, 2018) to generate random initializations of R’s internal Random Number Generator, which was set to the “Mersenne Twister” algorithm (cf. Matsumoto and Nishimura, 1998). To make the analyses based on random sampling exactly reproducible, we stored the random seeds as variables (for details, see the analysis scripts in the Supplementary Material).

The predictions vectors were set to contain probabilities. Summary metrics were computed based on average performance using 10-fold cross validation.

Performance tradeoff was evaluated using metrics based on the confusion matrix of model predictions and actual class membership according to the decoding test. Table 2 presents the confusion matrix and metrics that were considered. Both metrics indicating usefulness of the model for assessment of low decoding skill (i.e., positive predictive value [PP] and negative predictive value [NP]), and accurate identification of children with low decoding skill by the model (i.e., accuracy, sensitivity [SE], specificity [SP]) were considered. Usefulness of the model for assessment of low decoding skill concerns the likelihood that the constructed model can successfully identify whether children indeed have the lowest decoding skill or not, as illustrated by the last column of Table 2. Accurate identification of children with low decoding skill by the model concerns the similarity of the allocation of children to the lowest decoding group by the model to actual lowest decoding skill according to the word decoding test, as illustrated by the last row of Table 2 (Trevethan, 2017).

**TABLE 2:** Confusion matrix for positive class and negative class allocation by the models.

Group membership according to model	Group membership according to word decoding test		Performance statistic (usefulness)
	
	Lowest decoding	Not lowest decoding	
Lowest decoding	TP	FP	PP
Not lowest decoding	FN	TN	NP
Performance statistic (identification)	SE	SP	Accuracy

*TP, True positives; FP, False positives; FN, False negatives; TN, True negatives; PP, Positive predictive value; NP, Negative predictive value; SE, Sensitivity; SP, Specificity.*

##### Positive Predictive Value

The proportion of positive cases that were accurately allocated to the target category (lowest decoding level group) by the model, that is, when the model allocates members to the low decoding level group, how many belong to this group according to the word decoding test? PP = $\frac{\mathrm{true}\,\mathrm{positives}}{\mathrm{true}\,\mathrm{positives}+\mathrm{false}\,\mathrm{positives}}$

##### Negative Predictive Value

The proportion of negative cases that were accurately allocated to the non-target category (not lowest decoding level group), that is, when the model allocates members to the not-lowest decoding level group, how many belong to this group according to the word decoding test? NP = $\frac{\mathrm{true}\,\mathrm{negatives}}{\mathrm{true}\,\mathrm{negatives}+\mathrm{false}\,\mathrm{negatives}}$

##### Sensitivity

The proportion of cases that were accurately allocated to the target category (lowest decoding level group) by the model, that is, of the children who belong to the lowest decoding level group according to the test, how many were allocated to the lowest decoding group by the model? SE = $\frac{\mathrm{true}\,\mathrm{positives}}{\mathrm{true}\,\mathrm{positives}+\mathrm{false}\,\mathrm{negatives}}$

##### Specificity

The proportion of cases that were accurately allocated to the non-target category (not lowest decoding level group), that is, of the children who do not belong to the lowest decoding level group according to the test, how many were not allocated to the lowest decoding group by the model? SP = $\frac{\mathrm{true}\,\mathrm{negatives}}{\mathrm{true}\,\mathrm{negatives}+\mathrm{false}\,\mathrm{positives}}$

##### Accuracy

The proportion of cases that were accurately allocated to the non-target and to the target group. Accuracy = $\frac{\mathrm{true}\,\mathrm{positivestrue}\,\mathrm{negatives}}{\mathrm{true}\,\mathrm{positives}+\mathrm{true}\,\mathrm{negatives}+\mathrm{false}\,\mathrm{positives}+\mathrm{false}\,\mathrm{negatives}}$

##### Cohen’s Kappa (κ)

Statistic with values between 0 and 1, indicating the agreement between predictions of the model (decoding level within lowest decoding category or not) and the true membership of decoding level category (according to the word decoding test). The results of Cohen’s Kappa were evaluated to account for the possibility of accurate prediction by chance alone, which is an evident risk because of class imbalance in the present study. In the present study, Cohen’s κ will interpreted as suggested by Lantz (2019, p. 324):

• Poor agreement = less than 0.20
• Fair agreement = 0.20 to 0.40
• Moderate agreement = 0.40 to 0.60
• Good agreement = 0.60 to 0.80
• Very good agreement = 0.80 to 1.00

##### Receiver Operating Characteristic Curve

Visualization of the tradeoff between the sensitivity and the proportion of cases that were falsely allocated to the target category by the model.

##### Area Under the Curve

Statistics with values varying between 0.5 and 1, with higher values indicating better predictive models. AUC is based on the tradeoff between sensitivity and the proportion of cases that were falsely allocated to the target category by the model. Although some guidelines for classifying AUC are available, AUC is best evaluated in a comparative way. In the present study, AUC will be classified using the convention suggested by Lantz (2019, p. 333):

• No Discrimination = 0.5 to 0.6
• Poor = 0.6 to 0.7
• Acceptable / Fair = 0.7 to 0.8
• Excellent/Good = 0.8 to 0.9
• Outstanding = 0.9 to 1.0

Due to the fact that the binary classes were not evenly distributed (80% / 20%), some distortion of the metrics were expected. Specifically, the uneven distribution in favor of the non-target category leads to a higher chance of correct allocation to the non-target category. Therefore, results were interpreted primarily using Cohen’s Kappa, ROC curve, and AUC.

## Study 1

In this study, we built a model from an existing dataset on phonological abilities and word-decoding accuracy in Dutch children. The present database originates from research conducted by Verhoeven (Expertisecentrum Nederlands) and Keuning (Cito) on precursors of dyslexia in Dutch children (see Verhoeven and Keuning, 2018). Study 1 aimed at answering the question: To what extent are cognitive skills indicative of present decoding level? Baseline cognitive skills in Study 1 consisted of phonological awareness skills, rapid naming skills, and working memory skills (for a detailed description see Supplementary Material B).

### Materials and Methods

#### Participants

Participants were Dutch children attending 68 elementary schools: 782 in Grade 3, 707 in Grade 4, 263 in Grade 5, and 255 in Grade 6 after deletion of cases with missing variables.

#### Materials

Measures of one output variable and six input variables were collected using assessments. The output variable consisted of a test score for word-decoding efficiency and the input variables of test scores for nonword repetition, naming speed, phoneme segmentation, and phoneme deletion. A detailed description of the tests that were used can be found in the Supplementary Material B.

#### Procedure

Assessment of input and output variables took place in an individual setting by trained graduate students. The sequence of the tests within a session was randomized. All students were assessed halfway the school year. For more details about the methods, see Verhoeven and Keuning (2018).

### Results

Means and standard deviations for all input variables and the output variable of the models are presented in Table 3. The output variable (word-decoding efficiency) was transformed into binary classes, with 438 cases in the 20% lowest decoding level class and 1571 cases in the alternative class.

**TABLE 3:** Descriptive statistics for input variables and output variable of the models (*n* = 2009).

	*Range*	*M*	*SD*
**Input variables**			
Nonword repetition	11–40	33.72	4.77
Naming speed – digits	43–166	96.25	17.09
Naming speed – letters	1–157	91.79	19.13
Naming speed – pictures	5–129	61.82	11.93
Phoneme segmentation	0–20	18.55	3.07
Phoneme manipulation	0–20	18.55	2.52
**Output variable**			
Word decoding efficiency	0.5–120.75	57.47	20.88

Evaluation results of the models built with five machine learning techniques are presented in Table 4. Concerning identification of low decoding skill by the models, Positive Predictive (PP) and Negative Predictive (NP) values were evaluated. The results on the 95% confidence-intervals of PP appear between 0.25 and 0.37. Thus, between 25 and 37% of children allocated to the low decoding-category by the model, truly performed within the low decoding category when assessed with a decoding test; 63 to 75% of children allocated to the low decoding-category did not. The results on the 95% confidence-intervals of NP revealed that 92 to 96% of children not allocated to the low decoding-category by the model, truly did not perform within the low decoding category when assessed with a decoding test; 4 to 8% of children not allocated to the low decoding-category did.

**TABLE 4:** Confidence intervals of summary statistics for the predictive ability of the models built with five machine learning techniques.

Technique	PP 95% CI	NP 95% CI	SE 95% CI	SP 95% CI	Acc 95% CI	*κ*95% CI	AUC95% CI
Neural network	[0.32, 0.34]	[0.94, 0.95]	[0.61, 0.65]	[0.83, 0.84]	[0.81, 0.82]	[0.29, 0.34]	[0.77, 0.79]
K-nn	[0.27, 0.30]	[0.94, 0.95]	[0.58, 0.62]	[0.83, 0.83]	[0.80, 0.81]	[0.27, 0.30]	[0.73, 0.75]
Random Forests	[0.32, 0.35]	[0.93, 0.94]	[0.57, 0.61]	[0.83, 0.84]	[0.80, 0.81]	[0.30, 0.33]	[0.78, 0.79]
Xg-boost	[0.34, 0.37]	[0.92, 0.93]	[0.55, 0.59]	[0.84, 0.84]	[0.80, 0.81]	[0.31, 0.34]	[0.77, 0.79]
GLM	[0.25, 0.28]	[0.96, 0.96]	[0.64, 0.68]	[0.82, 0.83]	[0.81, 0.81]	[0.27, 0.30]	[0.77, 0.79]

*PP, positive predictive value; NP, negative predictive value; SE, sensitivity; SP, specificity; Acc, accuracy; κ, Kappa; AUC, area under the ROC; CI, confidence interval.*

Concerning the usefulness of the models of detecting low decoding skill, accuracy, sensitivity, and specificity were evaluated. The results of the 95% confidence-intervals of accuracy revealed that between 80 and 82% of the children were correctly allocated to the right decoding skill category by the models. The results on the 95% confidence-intervals of sensitivity showed that between 55 and 68% of children who truly performed within the low decoding category when assessed with a decoding test, were indeed allocated to the low decoding group by the model; 45% to 32% of children with actual low decoding skills were not. The results on the 95% confidence-intervals of specificity revealed that between 82 and 84% of children who truly performed not within the low decoding-category when assessed with a decoding test, were indeed allocated to the not low decoding group by the model; 16 to 18% of children without actual low decoding skill were falsely allocated to the low decoding group by the model. The results of Cohen’s κ were evaluated to account for the possibility of accurate prediction by chance alone. The results on the 95% confidence-intervals of the Cohen’s κ appear between 0.27 and 0.34, and indicate fair agreement between the models’ predictions and the true values. See “Materials and Methods” section for suggested interpretation of Cohen’s κ.

**FIGURE 2:**
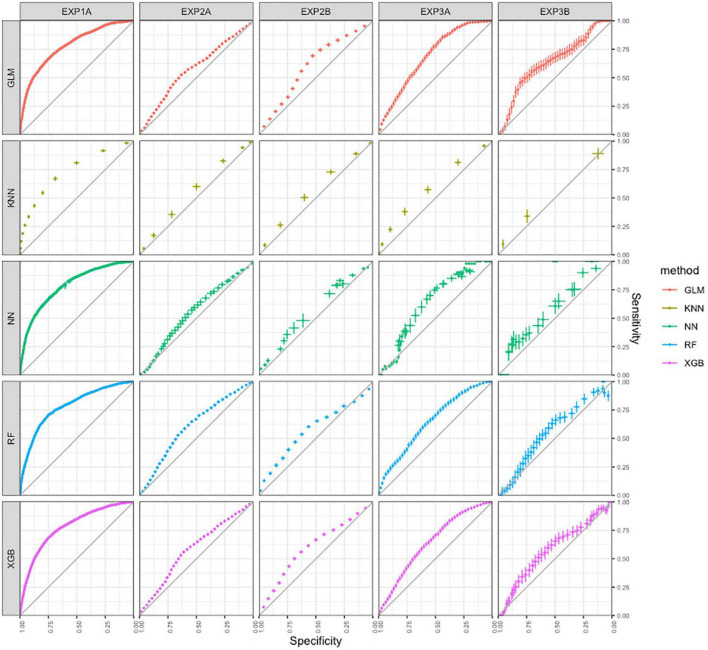
ROC-curves for the predictive ability of the models in all three studies built with five machine learning techniques.

The charts in the first column of Figure 2 visualize results of identification of first-quintile word-decoding efficiency for all models. The curves indicate positive predictive abilities of all models. The results on the 95% confidence-intervals of the AUC-statistics appear between 0.73 and 0.79, and indicate acceptable identification of first quintile word decoding for all models. See “Materials and Methods” section for suggested interpretation of AUC statistics. Visual inspection of the curves confirms the similarity between the five models pertaining to results of the AUC statistic.

### Conclusion

Study 1 aimed at building a model of five cognitive skills input variables to predict which participants achieved within the 20th percentile of decoding skill of children attending regular Dutch schools. The results indicated that model building by nonlinear machine learning techniques yields results comparable to model building by more traditional linear techniques. The predictive ability of both the linear model and the four nonlinear models appeared to be acceptable, as indicated by the ROC curves and AUC statistics. Balancing the tradeoff between sensitivity and specificity reveals best results in terms of specificity, at the expense of sensitivity, that is, the models tend to identify about 80% of children without decoding problems correctly (specificity), but only about 60% of children who indeed have low decoding skill (sensitivity), which results in accuracy of about 80%. The results of Cohen’s κ, however, indicate only fair agreement between the expected decoding category and the true decoding category. Recall that accuracy can be overestimated due to class imbalance, which is the case in the present study (see “General Methods” section). The weak agreement according to Cohen’s κ demonstrates that accuracy scores were affected by the imbalanced categories.

## Study 2

In this study, we built a model from a dataset on performance on cognitive tests and word-decoding accuracy in Dutch children with dyslexia. Two types of model design were evaluated: Study 2a models fourteen cognitive predictors and uses decoding accuracy as an outcome variable at the same moment of measurement, pertaining to the question: To what extent are cognitive skills indicative of present decoding level? Study 2b models fifteen cognitive predictors at T1 and uses decoding accuracy after three months of reading and spelling remediation (T2) as an outcome variable, pertaining to the question: To what extent are cognitive skills predictive of future decoding progress? Baseline cognitive skills in Study 2 consisted of phonological awareness skills, rapid naming skills, working memory skills, nonverbal reasoning skills, and vocabulary skills (for a detailed description, see Supplementary Material B).

### Materials and Methods

#### Participants

After excluding all cases with missing variables, the data of 383 children attending Braams & Partners (a Dutch clinic for the assessment and remediation of learning disorders) could be used in this study. All participants were attending Dutch primary schools Grade 2–6 and were diagnosed with severe dyslexia according to the criteria of Blomert (2006) and the Stichting Dyslexie Nederland (Dutch Dyslexia Foundation).

#### Materials

Measures of one output variable and fourteen input variables were collected using the following assessments. The output variable consisted of a test score for word decoding. The input variables consisted of test scores for grapheme-phoneme identification, grapheme-phoneme discrimination, naming speed, vocabulary, nonverbal reasoning, digit recall, block recall, and word recall. A detailed description of the tests that were used can be found in the Supplementary Material B.

#### Procedure

The tests administered at the clinic assessed whether criteria were met for severe dyslexia and thus for reading and spelling remediation at the clinic. Only children who met the criteria for severe dyslexia were included in the study. The Protocol Dyslexia Diagnosis and Treatment (Blomert, 2006) required the following criteria for a diagnosis of severe singular dyslexia:

(1) Persistence: The main criterion for referral to a clinic was that students appear not to profit from at least eight weeks of extra reading and spelling remediation in school, which is roughly operationalized in persisting scores below the 10th percentile on reading tests.
(2) Severity: Upon referral, severity of the reading and spelling impairment was assessed by means of several standardized reading and spelling tests.
(3) Cog0itive profile: Other cognitive skills associated with dyslexia (e.g., phonological processing, rapid naming, verbal working memory) were assessed as well.
(4) Differential diagnostics: To exclude students whose reading and spelling impairment stem from alternative “causes,” several additional cognitive skills were assessed (e.g., IQ, nonverbal memory). Children with other diagnoses that can account for language problems, such as SLI and sensory problems, were also excluded.

Once assessment results met all criteria, the student was diagnosed with severe singular dyslexia and was eligible for a subsidized, specialized reading and spelling remediation program at the clinic. Students who did not satisfy these criteria were provided with specific recommendations for remediation in school or referred to another specialist, depending on assessment results.

### Results

Means and standard deviations for all input variables and the output variable of the models are presented in Table 5. The output variable (word decoding efficiency) was transformed into binary classes, with 190 cases in the lowest decoding level class and 194 cases in the alternative class in Study 2a and 99 cases in the lowest decoding level class and 84 cases in the alternative class in Study 2b.

**TABLE 5:** Descriptive statistics for input variables and output variable of the models.

Variable	Study 2a *(n = 384)*			Study 2b *(n = 183)*		
		
	*Range*	*M*	*SD*	*Range*	*M*	*SD*
**Input variables**						
Grapheme-phoneme identification						
Speed	1.28–5.60	2.54	0.58	1.28–4.99	2.58	0.57
Accuracy	35.56–100	87.26	8.66	53.33–100.00	86.61	8.19
**Grapheme-phoneme discrimination**						
Speed	0.83–19.44	1.85	1.04	0.00–3.39	1.80	0.52
Accuracy	17.78–96.67	81.39	10.02	0.98–95.56	80.40	11.74
**Naming speed**						
Digits	3.54–42.58	10.39	3.03	6.51–42.58	10.57	3.45
Letters	3.78–20.63	11.25	2.53	7.14–19.28	11.32	2.37
Pictures	3.71–31.22	15.66	3.54	9.72–31.22	15.60	3.41
Vocabulary	57–144	110.11	9.96	85–144	110.84	9.26
Nonverbal reasoning	5–119	19.90	6.78	5–119	20.46	9.22
**Digit recall**						
Forward	6–36	24.15	3.83	11–32	24.25	3.92
Backward	3–39	11.05	3.74	5–30	11.17	3.65
Block recall	2–37	25.58	4.45	2–37	25.76	4.41
**Word recall**						
Reproduction	16–61	39.86	8.47	16–61	40.21	8.26
Recall	1–15	8.51	2.71	1–15	8.47	2.82
**Output variable**						
Word decoding efficiency	0–6	1.45	1.29	0–5	1.50	1.49
						

Evaluation results of the models built with five machine learning techniques are presented in Table 6 for Study 2a and in Table 7 for Study 2b. Concerning identification of lowest decoding skill by the models, PP and NP were evaluated. The results on the 95% confidence-intervals of PP reveal that between 33 and 56% of children allocated to the lowest decoding category by the model, truly performed within the lowest decoding category when assessed with a decoding test; 54 to 67% of children allocated to the low decoding-category did not. The results on the 95% confidence-intervals of NP reveal that between 53 and 71% of children not allocated to the lowest decoding category by the model, truly did not perform within the lowest decoding category when assessed with a decoding test; 29 to 47% of children not allocated to the lowest decoding category did.

**TABLE 6:** Confidence intervals of summary statistics for the predictive ability of the models built with five machine learning techniques in study 2a.

Technique	PP95% CI	NP95% CI	SE95% CI	SP95% CI	Acc95% CI	*κ*95% CI	AUC95% CI
Neural network	[0.33, 0.45]	[0.59, 0.71]	[0.52, 0.58]	[0.51, 0.54]	[0.51, 0.53]	[0.02, 0.06]	[0.55, 0.57]
K-nn	[0.50, 0.54]	[0.53, 0.57]	[0.52, 0.55]	[0.52, 0.55]	[0.52, 0.55]	[0.04, 0.09]	[0.56, 0.58]
Random forests	[0.33, 0.45]	[0.59, 0.71]	[0.52, 0.58]	[0.51, 0.54]	[0.51, 0.53]	[0.02, 0.06]	[0.60, 0.63]
Xg-boost	[0.52, 0.56]	[0.57, 0.61]	[0.55, 0.59]	[0.55, 0.57]	[0.55, 0.58]	[0.10, 0.15]	[0.57, 0.60]
GLM	[0.48, 0.53]	[0.58, 0.62]	[0.54, 0.58]	[0.53, 0.56]	[0.54, 0.57]	[0.07, 0.13]	[0.56, 0.59]

*PP, positive predictive value; NP, negative predictive value; SE, sensitivity; SP, specificity; Acc, accuracy; κ, Kappa; AUC, area under the ROC; CI, confidence interval.*

**TABLE 7:** Confidence intervals of summary statistics for the predictive ability of the models built with five machine learning techniques in study 2b.

Technique	PP95% CI	NP95% CI	SE95% CI	SP95% CI	Acc95% CI	*κ* 95% CI	AUC95% CI
Neural network	[0.70, 0.79]	[0.20, 0.27]	[0.51, 0.53]	[0.42, 0.53]	[0.49, 0.53]	[–0.05, 0.02]	[0.54, 0.58]
K-nn	[0.59, 0.66]	[0.34, 0.40]	[0.51, 0.54]	[0.44, 0.50]	[0.48, 0.53]	[–0.05, 0.04]	[0.56, 0.60]
Random forests	[0.53, 0.60]	[0.37, 0.45]	[0.50, 0.54]	[0.42, 0.48]	[0.47, 0.51]	[–0.07, 0.02]	[0.56, 0.60]
Xg-boost	[0.48, 0.55]	[0.38, 0.44]	[0.47, 0.52]	[0.40, 0.46]	[0.44, 0.49]	[–0.12, –0.03]	[0.58, 0.62]
GLM	[0.58, 0.64]	[0.34, 0.41]	[0.50, 0.54]	[0.42, 0.49]	[0.47, 0.52]	[–0.07, 0.03]	[0.59, 0.63]

*PP, positive predictive value; NP, negative predictive value; SE, sensitivity; SP, specificity; Acc, accuracy; κ, Kappa; AUC, area under the ROC; CI, confidence interval.*

Concerning the usefulness of the models to detect lowest decoding skill, accuracy, sensitivity, and specificity are evaluated. The results of the 95% confidence-intervals of accuracy reveal that between 51 and 58 % of the children were correctly allocated to the right decoding skill category by the models. The results on the 95% confidence-intervals of sensitivity reveal that between 52 and 59% of children who truly performed within the lowest decoding category when assessed with a decoding test, were indeed allocated to the lowest decoding group by the model; 41 to 48% of children with actual lowest decoding skills were not. The results on the 95% confidence-intervals of specificity reveal that between 51 and 56% of children who truly performed not within the lowest decoding-category when assessed with a decoding test, were indeed allocated to the not lowest decoding group by the model; 44 to 49% of children without actual lowest decoding skill were falsely allocated to the lowest decoding group by the model. Cohen’s κ -were evaluated to account for the possibility of accurate prediction by chance alone. The results on the 95% confidence-intervals of the κ statistics appear between 0.02 and 0.15, and indicate poor agreement between the models’ predictions and the true values. See “Materials and Methods” section for suggested interpretation of Cohen’s κ.

The charts in the second column of Figure 2 visualize results on identification of the lowest decoding level class for all models in Study 2a, and the charts in de third column for Study 2b. For both studies, the curves indicate minimal yet predominantly positive predictive abilities of all models, with little to no differences between models based on different techniques. The results on the 95% confidence-intervals of the AUC-statistics appear between 0.55 and 0.63, and indicate no to poor identification of lowest word decoding for all models. See “Materials and Methods” Section for suggested interpretation of AUC statistics. Visual inspection of the curves confirms the similarity between the five models on results on the AUC statistic.

### Conclusion

Study 2 was aimed at building a model of fourteen cognitive skills input variables to predict which participants achieved within the lowest category of decoding skill within children with severe dyslexia. The results of this study again indicated that results on model building by nonlinear machine learning techniques are comparable to results on model building by more traditional linear techniques. The predictive ability of both the linear model and the four nonlinear models appeared to be poor, as indicated by the ROC curves and AUC statistic. Balancing the tradeoff between sensitivity and specificity indicates that the models tend to identify only about 50% of children without lowest decoding skill correctly (specificity), and only about 50% of children who have indeed lowest decoding skill (sensitivity), which results in a disappointing accuracy of about 50%. Correcting for the imbalanced categories, the results of Cohen’s κ leave only poor agreement between the expected decoding category and the true decoding category. Results for prediction of participants achieving within the lowest category of decoding skill at T1 (Study 2a, aimed at word decoding level) were comparable to those at T2 (Study 2b, aimed at word decoding progress).

## Study 3

In this study, the predictive validity of five phonological-awareness tests on initial reading was investigated in Dutch children attending primary school. Phonological awareness was assessed once: In the last year of kindergarten (T1). Word decoding was assessed twice: Halfway Grade 1 (T2, providing results for Study 3a) and at the end of Grade1 (T3, providing results for Study 3b), both answering to the question: To what extent are cognitive skills predictive of future decoding progress? Baseline cognitive skills in Study 3 consisted of phonological awareness skills (for a detailed description see Supplementary Material B).

### Materials and Methods

#### Participants

Participants were Dutch children attending three primary schools. The group that was assessed on phonological awareness halfway the last year of kindergarten (cohort 2) consisted of 121 participants, and the group that was assessed on phonological awareness at the end of the last year of Kindergarten (cohort 1) consisted of 82 participants, after deletion of cases with missing variables in Study 3a. In Study 3b, cohort 2 consisted of 82 participants, and cohort 1 consisted of 117 participants, after deletion of cases with missing variables.

#### Materials

Measures of one output variable and five input variables were collected using assessments. The measures of the input variables were designed by Braams (see Braams and Bosman, 2000). The output variable consisted of a test score for word decoding. The input variables consisted of test scores for rhyme, rhyme prime, auditory synthesis, phoneme deletion, and letter naming. A detailed description of the tests that were used can be found in Supplementary Material B.

#### Procedure

Participants were split in two groups: Cohort 1 and Cohort 2. Participants in both cohorts were assessed on word decoding twice in Grade 1, halfway and at the end of the schoolyear. Participants in Cohort 2 were assessed on the five phonological awareness measures halfway the last year in kindergarten, and participants in Cohort 1 at the end of the last year in Kindergarten. For more details about the methods, see Braams and Bosman (2000).

### Results

Means and standard deviations for all input variables and the output variable of the models are presented in Table 8. The output variable (word decoding efficiency) was transformed into binary classes, with 42 cases in 20% lowest decoding level class and 161 cases in the alternative class in Study 3a, and 48 cases in 20% lowest decoding level class and 151 cases in the alternative class in Study 3b. Scores of input variables were transformed into Z-scores, in order to even out effects of differing assessment moments between cohort 1 and 2.

**TABLE 8:** Descriptive statistics for raw scores on input variables and output variable of the model.

Variable	Study 3a *(n = 203)*			Study 3b *(n = 199)*		
		
	*range*	*M*	*SD*	*range*	*M*	*SD*
**Input variables**						
Rhyme	0–28	17.67	5.11	0–28	17.75	4.96
Rhyme prime	2–26	12.54	5.05	2–26	12.54	5.04
Auditory synthesis	0–24	13.42	4.98	0–24	13.43	4.98
Phoneme deletion	0–29	12.47	7.98	0–29	12.41	7.98
Letter naming	0–20	7.71	6.52	0–20	7.73	6.49
**Output variable**						
Word decoding efficiency	4–68	16.16	10.39	4–74	27.32	14.16

Evaluation results of the models built with five machine learning techniques are presented in Tables 9, 10. Concerning identification of low decoding skill by the models, PP and NP were evaluated. The results on the 95% confidence-intervals of PP reveal that between 8% and 25% of children allocated to the low decoding-category by the model, truly performed within the low decoding category when assessed with a decoding test; 75 to 92% of children allocated to the low decoding-category did not. The results on the 95% confidence-intervals of NP reveal that between 88 and 98% of children not allocated to the low decoding-category by the model, truly did not perform within the low decoding category when assessed with a decoding test; 2 to 12% of children not allocated to the low decoding-category did.

**TABLE 9:** Confidence intervals of summary statistics for the predictive ability of the models built with five machine learning techniques in study 3a.

Technique	PP95% CI	NP95% CI	SE95% CI	SP95% CI	Acc95% CI	*κ* 95% CI	AUC95% CI
Neural network	[0.10, 0.15]	[0.88, 0.93]	[0.20, 0.30]	[0.80, 0.80]	[0.79, 0.80]	[0.00, 0.01]	[0.64, 0.66]
K-nn	[0.13, 0.17]	[0.91, 0.96]	[0.41, 0.53]	[0.81, 0.82]	[0.78, 0.80]	[0.10, 0.15]	[0.60, 0.63]
Random Forests	[0.20, 0.25]	[0.88, 0.93]	[0.39, 0.46]	[0.82, 0.83]	[0.77, 0.79]	[0.14, 0.20]	[0.63, 0.67]
Xg-boost	[0.17, 0.22]	[0.84, 0.90]	[0.28, 0.35]	[0.81, 0.82]	[0.74, 0.76]	[0.07, 0.13]	[0.63, 0.66]
GLM	[0.08, 0.11]	[0.94, 0.98]	[0.42, 0.57]	[0.81, 0.81]	[0.79, 0.80]	[0.05, 0.10]	[0.67, 0.71]

*PP, positive predictive value; NP, negative predictive value; SE, sensitivity; SP, specificity; Acc, accuracy; κ, Kappa; AUC, area under the ROC; CI, confidence interval.*

**TABLE 10:** Confidence intervals of summary statistics for the predictive ability of the models built with five machine learning techniques in study 3b.

Technique	PP95% CI	NP95% CI	SE95% CI	SP95% CI	Acc95% CI	*κ* 95% CI	AUC95% CI
Neural network	[0.36, 0.43]	[0.72, 0.80]	[0.40, 0.49]	[0.75, 0.76]	[0.75, 0.76]	[0.00, 0.01]	[0.65, 0.67]
K-nn	[0.20, 0.24]	[0.91, 0.93]	[0.48, 0.58]	[0.78, 0.79]	[0.74, 0.76]	[0.14, 0.19]	[0.63, 0.66]
Random Forests	[0.19, 0.23]	[0.91, 0.93]	[0.46, 0.56]	[0.78, 0.79]	[0.74, 0.76]	[0.14, 0.19]	[0.61, 0.64]
Xg-boost	[0.23, 0.28]	[0.84, 0.87]	[0.35, 0.41]	[0.78, 0.79]	[0.70, 0.72]	[0.10, 0.15]	[0.61, 0.64]
GLM	[0.07, 0.10]	[0.93, 0.95]	[0.28, 0.40]	[0.76, 0.76]	[0.73, 0.74]	[0.01, 0.05]	[0.67, 0.70]

*PP, positive predictive value; NP, negative predictive value; SE, sensitivity; SP, specificity; Acc, accuracy; κ, Kappa; AUC, area under the ROC; CI, confidence interval.*

Concerning the usefulness of the models to detect low decoding skill, accuracy, sensitivity, and specificity were evaluated. The results of the 95% confidence-intervals of accuracy reveal that between 74 and 80% of the children were correctly allocated to the right decoding skill category by the models. The results on the 95% confidence-intervals of sensitivity reveal that between 20 and 57% of children who truly performed within the low decoding category when assessed with a decoding test, were indeed allocated to the low decoding group by the model; 43 to 80% of children with actual low decoding skills were not. The results on the 95% confidence-intervals of specificity reveal that between 80 and 83% of children who truly performed not within the low decoding-category when assessed with a decoding test, were indeed allocated to the not low decoding group by the model; 17 to 20% of children without actual low decoding skill were falsely allocated to the low decoding group by the model. The results of Cohen’s κ -were evaluated to account for the possibility of accurate prediction by chance alone, which is an evident risk because of class imbalance in the present study. The results on the 95% confidence-intervals of Cohen’s κ appear between 0.00 and 0.15, and indicate poor agreement between the models’ predictions and the true values. See “Materials and Methods” Section for suggested interpretation of Cohen’s κ.

The charts in the fourth column of Figure 2 visualize results on identification of the 20% lowest decoding level class for all models in Study 3a, and the charts in de fifth column for Study 2b. For both studies, the curves indicate minimal yet predominantly positive predictive abilities of all models, with little to no differences between models based on different techniques. The results on the 95% confidence-intervals of the AUC-statistics appear between 0.60 and 0.67, and indicate poor identification of first quintile word decoding for all models. See “Materials and Methods” Section for suggested interpretation of AUC statistics. Visual inspection of the curves confirms the similarity between the five models on results on the AUC statistic.

### Conclusion

Study 3 was aimed at building a model of five cognitive skills input variables to predict which participants achieved within the lowest 20th percentile of decoding skills among children attending Grade 1 of regular Dutch education. The results again indicated that results on model building by nonlinear machine learning techniques are comparable to results on model building by more traditional linear techniques. The predictive ability of both the linear model and the four nonlinear models appeared to be poor, as indicated by the ROC curves and AUC statistics. Balancing the tradeoff between sensitivity and specificity reveals best results in terms of specificity, at the expense of sensitivity, that is, the models tend to identify about 80% of children without decoding problems correctly (specificity), but only about 20–50% of children who have indeed low decoding skill (sensitivity), which results in accuracy of about 75%. The results of Cohen’s κ, however, indicate poor agreement between the expected decoding category and the true decoding category, demonstrating that accuracy scores were affected by the imbalanced categories. Results for prediction of participants achieving within the lowest 20th percentile of decoding skills at T2 (Study 3a) were comparable to those at T3 (Study 3b).

## General Discussion

The main goal of the present study was to find whether cognitive factors of varying origin play any role in the development of reading skill in children with dyslexia and children with typical reading development. The present study focused on the possibilities of nonlinear machine learning techniques compared to traditional, linear statistical techniques.

The results of the present study pertain to two research questions: (1) To what extent are cognitive skills indicative of present decoding difficulties (Studies 1 and 2a), and (2) To what extent are cognitive skills predictive of future decoding difficulties yielded similar findings (Studies 2b, 3a, and 3b). Results of models built with nonlinear machine learning techniques were comparable to results of models built by the more traditional linear (GLM) technique and results on data of average school-aged children were comparable to those on data of children with severe dyslexia.

### Cognitive Skills and Decoding Level

Irrespective of the building technique, models built to determine *present* decoding skill level based on an input set of *present* cognitive skills seem to produce the best results in the present study. However, these results indicate an inadequate base for making a potential thorough prediction of reading development. The best performing models in the present study only produced an AUC 95%-confidence interval of 0.78–0.79, corresponding to a proportion explained variance of 0.23–0.25 (see Ruscio, 2008). Leaving 75% of variance unexplained, these results are comparable to the results on correlations between cognitive skills and reading skill discussed in the “Introduction” section (Hammill and McNutt, 1981; Scarborough, 1998; Swanson et al., 2003; Melby-Lervåg, 2012).

These results are in line with those of Verhoeven and Keuning (2018), who studied the predictive ability of several models on the dataset used for Study 1 of the present study. The present study differed from the study of Verhoeven and Keuning (2018) in two ways: (1) Verhoeven and Keuning (2018) used the logistic regression analyses (based on linear techniques, and using traditional statistical analysis for data modeling) to build the models, whereas the present study used several techniques (linear and nonlinear) of machine learning, and (2) Verhoeven and Keuning (2018) aimed to predict a dyslexia diagnosis (according to formal criteria of the Dutch Dyslexia Foundation) as the outcome variable, whereas the present study aimed to predict low decoding skill. Verhoeven and Keuning (2018) found an AUC of 0.84 when modeling cognitive skills variables on dyslexia diagnosis, which roughly agrees with the results of the AUC 95%-confidence intervals of best performing models in the present study (AUC 0.78–0.79, see Table 4 in “Results” section). A question arises when we look at the outcome variables, which differed between these particular studies, as noted earlier. In our study, the variable of the dyslexia diagnosis was only to a limited extent related with low decoding skill (only 38% of dyslexic children belonged to the target category “decoding level within lowest 20%”) in the dataset of Verhoeven and Keuning (2018). This suggests that the children in their sample with a dyslexia diagnosis are not necessarily performing worst on decoding skill. Thus, irrespective of using traditional statistical analyses versus machine learning techniques for data modeling, and irrespective of the outcome being low word decoding or a dyslexia diagnosis, these studies on the role of cognitive skills in the field of reading ability suggest that relationships between cognitive skills and reading ability do exist. The relative weakness of this relationship does not justify the conclusion that these skills play a substantial role in reading performance. Therefore, cognitive skills and decoding skill are *moderately related*, leaving room for considering (1) other potential variables that could be related to reading skill, and (2) more complicated relationships than only the unidirectional explanation of cognitive skills being predictive for decoding skill.

### Cognitive Skills and Decoding Progress

As Vellutino et al. (2004) pointed out, in order to predict future reading development, a model needs to include a variable that indicates progress in reading skill, which requires at least two moments of measurement. Studies 2b and 3b partly fulfilled this criterion, by modeling input cognitive variables on future decoding skill, corresponding to the unconditional models discussed in the “Introduction” section.

Models built to indicate future low decoding skill based on an input set of *present* cognitive skills seem to produce both weak and highly varying results in Studies 2b and 3b, which poses doubts on the usefulness of these models. A small caveat has to be made because the relatively low sample size may have troubled the prospects of building adequate models. Results of the present study have yet to be confirmed by future research to strengthen conclusions.

The results on the role of cognitive skills in reading progress of the present study are in line with research based on linear techniques, and using traditional statistical analysis for data modeling. The present study confirms results of correlational meta-analyses discussed in the “Introduction” section, that revealed no evident role of cognitive skills in reading development (Nelson et al., 2003; Tran et al., 2011). Furthermore, the present study confirms correlational studies on decoding skill and cognitive skills in children with dyslexia. Walda et al. (2014) found many significant (80%) correlations between present cognitive skills (e.g., executive functions) and present decoding level, whereas significant correlations between present cognitive skills and progress in decoding skill were nearly absent (8.2%). Walda et al. (2022) studied the role of attentional skill in children with dyslexia and found that the working speed component of attentional skill was related to decoding level, whereas both the working speed component and the distraction component of attentional skill did not affect progress in decoding skill during remediation.

Thus, present and past research suggest that it is unlikely that cognitive skills play a determinant role in future decoding progress. Previous research based on traditional statistical methods discussed in the “Introduction” section revealed unconvincing and unequivocal results. Results of the present study, based on machine learning techniques, although preliminary, confirms results of previous studies. As machine learning techniques enable the model to take into account multidirectional, reciprocal, and concurrent relationships between variables, results of the present study add to previous studies the cautious suggestion that a determinant role for cognitive skills in decoding progress is not evident.

### Implications for Future Research and Educational Practice

Apart from low sample size, some other factors may have flawed the potential of the used algorithms for model building. In Study 2, the sample included only children with dyslexia might have caused restriction of range in both input and output variables, as it is known that children with dyslexia tend to have lower abilities in both cognitive skills and decoding skill compared to average school aged children. Therefore, results of Study 2 should not be interpreted without considering results of Studies 1 and 3. Also, the output variable (decoding skill) was recoded in a binary variable with uneven classes (i.e., approximately 20–80% in study 1 and 3, and approximately 50%–50% in Study 2), which might have produced a slight class-imbalance problem, including only a small number of cases in the target category of the test set, especially when small samples are used.

In the present study, we have used several machine learning techniques to compare the results. Although different techniques differ in the extent to which they are sensitive to class imbalance, this problem seems limited because various techniques agree on the results. This was the case for Studies 1 and 2. In Study 3, however, the results did differ between the techniques that were used. We have tried to limit this problem in Study 3 by expanding the test set to 25% of the sample (instead of 10% in Studies 1 and 2) and by training and testing all models on 100 different seeds of the sample.

Our findings should be considered preliminary due to the fact that previous research using nonlinear techniques and adequate modeling in this field is scarce. We, therefore, suggest future research on the role of cognitive factors in reading development applying nonlinear techniques for model building and to include designs and variables that expand insights in development of future reading skill, as was suggested by Vellutino et al. (2004) and Stuebing et al. (2015). Still, adequate model building is complicated because it requires sufficient sample sizes and longitudinal data gathering on large sets of variables, producing high costs, dedicated and often long-lasting participation of many stakeholders, and time-consuming strategies. Such research on the specific group of children with learning disabilities is even more complicated: Because of limited prevalence, sampling a large number of children with dyslexia is problematic. Pending more substantial results in future research, we would like to propose some thoughts on practical implications of present results.

From a theoretical perspective, the present results reveal that cognitive skills do correlate with reading skill, suggesting a relationship. The nature of this relationship is, however, unclear and it is as yet unlikely that cognitive skills will make good predictions about reading skill.

From a prevention perspective, it seems unlikely that children at risk for reading difficulties could be identified by assessment of cognitive skills, even if these cognitive skills consist of phonological awareness (specifically the case in Studies 1 and 3). In this line, it seems not sensible to use results of individual cognitive skills assessment in identifying children eligible for a dyslexia diagnosis. Instead, individual reading development, operationalized by repeated assessment of (word and text) decoding skills, should make better predictions of longitudinal reading development. Fortunately, in the Netherlands the Protocol Dyslexie Diagnose en Behandeling version 3.0 [Protocol Dyslexia Diagnosis and Treatment] (Tijms et al., 2021) has dropped cognitive skills criteria as requirements to diagnose dyslexia and specifies solely criteria on the reading and writing skill level. Still, the Protocol Dyslexie Diagnose en Behandeling version 3.0 considers cognitive skills as protective and/or risk factors for developing reading disorder. However, pending more effective strategies to identify these children, it seems even more tenable to monitor initial literacy development in an intensive and professional way, and intervene immediately when potential struggles appear in any child (independent of a dyslexia diagnosis). It is noteworthy that the nature of machine learning techniques does not allow to identify which cognitive variables could be relevant from the prevention perspective, and we certainly do not want to fall into the trap of detailed interpretations about individual variables involved in a black box model (e.g., see Rudin, 2019). A slight drawback of machine learning techniques is that the mechanisms of the results that were produced are difficult to interpret (Lantz, 2019). Note, however, if there is any predictive value of cognitive factors regarding reading development, machine learning techniques should be able to at least identify them. The fact that none of the models in the present study had sufficient predictive value for decoding skill, seems to suggest that these cognitive skills have no valuable role in reading development from the prevention perspective.

In a similar way, from a remediation perspective, it seems unlikely that training cognitive skills could prevent children from developing reading difficulties or that training cognitive skills could stimulate reading development. In this line, we agree with Hammill (2004), that:

(1)professionals interested in improving literacy skills should focus on teaching written language abilities such as print awareness and book handling, letters, phoneme-letter correspondences, word recognition, alphabet knowledge, and comprehension and (2) the current interest in the role of nonprint abilities in reading such as phonological awareness, rapid naming, intelligence, and memory might be overemphasized (p. 453).

Moreover, awaiting results from future research suggested earlier, we propose to reconsider the role of cognitive skills in criteria for dyslexia or severe reading problems. If a lack of cognitive skills does prove to have accidental side effects in some but not all children with low reading skill, assessing cognitive skills does not have any use in diagnosis and selection, let alone remediation, of children who need extra attention. This is in accordance with a proposal of McEaneaney et al. (2006) to move away from defining disabilities relying on factors within individuals and to center instructional needs and future steps in the process of remediation, and with Vellutino et al. (2004), who argue that inadequate instruction and other experiential factors play a crucial role for many children developing reading difficulties, and that cognitive factors such as IQ should have less emphasis in diagnosing reading difficulties. Thus, both assessment and instruction of children who are learning to read should be focused on reading skill itself.

## Data Availability Statement

The datasets presented in this study can be found in online repositories. The names of the repository/repositories and accession number(s) can be found below: study 2: https://doi.org/10.17026/dans-2cq-96v9; study 3: https://doi.org/10.17026/dans-xun-4v56.

## Ethics Statement

Ethical review and approval was not required for the study on human participants in accordance with the local legislation and institutional requirements. Written informed consent to participate in this study was provided by the participants’ legal guardian/next of kin.

## Author Contributions

AB and SW contributed to conception and design of the study. SW organized the database and wrote the first draft of the manuscript. SW and FH performed the statistical analyses. All authors contributed to manuscript revision, read, and approved the submitted version.

## Conflict of Interest

The authors declare that the research was conducted in the absence of any commercial or financial relationships that could be construed as a potential conflict of interest.

## Publisher’s Note

All claims expressed in this article are solely those of the authors and do not necessarily represent those of their affiliated organizations, or those of the publisher, the editors and the reviewers. Any product that may be evaluated in this article, or claim that may be made by its manufacturer, is not guaranteed or endorsed by the publisher.
