# Supplementary material for: Identifying Determinants of Dyslexia: An Ultimate Attempt Using Machine Learning
Source: Front Psychol. 2022 Apr 7;13:869352. doi: 10.3389/fpsyg.2022.869352 (PMC9025592; doi:10.3389/fpsyg.2022.869352)
Supplement: Supplementary file 1 [file Data_Sheet_1.DOCX]

**Supplementary material A**

**Details about building, tuning, training, and testing the models**

***Neural networks.*** Models were built using nnet package, with the rprop+ algorithm, SSE error function, and Sigmoid activation function applied to the hidden neurons (not to the output neuron). The number of hidden layers was limited to 1 for restraint purposes and decay was set to vary between 0 and 0.0001.

***K-nn.*** Models were built using the caret package. The number of k was set to vary between 5, 7, 9 and 11. The number of k appeared to be 11 for the best fit models in Study 1, and varied between 5 and 11 for the best fit models in all other studies (with a median number of k of 9, except for Study 2b, which produced a median number of k of 11 for best fit models).

***Random forests.*** The mtry parameter was set to vary between 2, 4, and 6. Models were built using mtry 2 in Study 1, and 2 - 6 in Studies 2a, 2b, 3a, and 3b. The mtry parameter median appeared to be 2 for the best fit models in Studies 1, 2a, and 2b. The mtry parameter median for the best fit models appeared to be 4 in Studies 3a, and 2 in Study 3b.

***Xg-boost.*** Models were built using the xgboost package, with a maximum of 50 iterations. The learning rate parameter (eta) was set to vary between .5 and .7, and the minimum loss reduction parameter (gamma) was set to vary between .25, .50 and .75. Maximal depth of individual trees was set to vary between 3 and 6. The learning rate parameter appeared to be 0.5 for the best fit models in Study 1, and varied between 0.5-0.7 in Studies 2a, 2b, 3a, and 3b. The minimum loss reduction parameter appeared to be 0.75 for the best fit models in Study 1, and 0.25-0.75 in Studies 2a, 2b, 3a, and 3b. Maximal depth of individual trees was 3 for the best fit models in study 1, and varied between 3 and 6 for Studies 2a, 2b, 3a, and 3b (with a median of 6 for all, except for Studies 3a and 3b, which produced median maximal depths of 3).

***General linear model.*** Models were built using the caret package.
