# Supplementary material for: Identifying Determinants of Dyslexia: An Ultimate Attempt Using Machine Learning
Source: Front Psychol. 2022 Apr 7;13:869352. doi: 10.3389/fpsyg.2022.869352 (PMC9025592; doi:10.3389/fpsyg.2022.869352)
Supplement: Supplementary file 2 [file Data_Sheet_2.DOCX]

**Supplementary material B**

**Detailed description of materials in study 1, 2 and 3**

**Study 1**

**Nonword repetition**

This input variable was assessed using the Nonword Repetition Test (Verhoeven et al., 2013). Participants were requested to repeat 40 nonwords and the measure consisted of the number of correctly repeated items.

**Naming speed**

These input variables consisted of three types of stimuli: Letters, digits, and pictures, each presented on a separate card with five recurring items. Participants were requested to name as many items as possible in one minute per card, resulting in three measures of correctly named letters, digits, and pictures.

**Phoneme segmentation**

This input variable was assessed using 20 orally presented words of varying phonemic structure. Participants were requested to segment each word into its constituent sounds. The measure consisted of the number of correctly segmented items.

**Phoneme deletion**

This input variable was assessed using 20 orally presented words. Participants were requested to pronounce the resulting word when deleting the initial, second, prefinal or final sound of the word. The measure consisted of the number of correctly pronounced items.

**Word decoding efficiency**

This output variable was assessed using four word decoding cards of increasing orthographic complexity. Participants were requested to decode as many words as possible in one minute per card. The measure consisted of the average amount of words decoded correctly on the four cards.

**Study 2**

**Grapheme phoneme identification**

These input variables consisted of two measures of the subtest “grapheme phoneme identification” of Differential Diagnostics of Dyslexia [letter-klank identificatie, Differentiaal Diagnostiek van Dyslexie, 3DM] (Blomert & Vaessen, 2009)*.* The participant was visually presented with four graphemes on the computer screen and auditory with the sound of a phoneme and was asked to press the button corresponding to the grapheme that was mapping to the presented phoneme. The test consisted of 45 items. This assessment resulted in an accuracy measure (percentage of correct responses) and in a speed measure (average speed per item, in seconds).

**Grapheme phoneme discrimination**

These input variables consisted of two measures of the subtest “grapheme phoneme discrimination” of Differential Diagnostics of Dyslexia [letter-klank discriminatie, Differentiaal Diagnostiek van Dyslexie, 3DM] (Blomert & Vaessen, 2009)*.* The participant was visually presented with a grapheme on the computer screen and auditorily with the sound of a phoneme and was asked if the grapheme-phoneme mapping was correct or not by clicking the green or the red button on the button box. The test consisted of 90 items. This assessment resulted in an accuracy measure (percentage of correct responses) and in a speed measure (average speed per item, in seconds).

**Naming speed**

These input variables consisted of three subtests of Differential Diagnostics of Dyslexia [benoemsnelheid, Differentiaal Diagnostiek van Dyslexie, 3DM] (Blomert & Vaessen, 2009)*.* The participant was asked to name as quickly as possible letters, digits, and pictures respectively. Stimuli were presented on the computer screen. For each stimulus, a set of fifteen items was presented at a time, containing five different recurring items. Each stimulus was presented twice, resulting in average reaction times for the total of two letter tasks, two-digit tasks, and two picture tasks.

**Vocabulary**

This input variable was assessed with the Dutch version of the Peabody Picture Vocabulary Test-III (PPVT-III-NL; Schlichting, 2005). The participant is presented visually with sheets containing four pictures. The participant is asked to pick the right picture corresponding to the auditory presented word. Dependent on participant’s age, a particular starting set of appropriate level is presented, and depending on performance on each subsequent set, the test is either aborted or continued by a set of increasing difficulty. Each set contained twelve items, and the total test contained 17 sets. However, none of the participants was presented all 17 sets because of the starting set and abortion rule. The measure consisted of the total number of correct responses (including the items prior to the starting set, which were considered as known).

**Nonverbal reasoning**

This input variable was assessed with the Matrix reasoning subtest of Wechsler’s nonverbal scale of ability (WNV-NL; Wechsler & Naglieri, 2008). The test assesses processes of perceptual reasoning and the simultaneous processing of information. In this test participants had to evaluate how different shapes and geometric elements relate to the target spatially or logically. They had to complete the pattern by choosing a shape from four to six options. Dependent on participant’s age, a particular starting item of appropriate level is presented, and the test is aborted when the participant failed on four out of five consecutive items. The maximum number of presented items was 41. The measure consisted of the number of correct responses.

**Digit recall**

These input variables consisted of two subtests of the Working memory test battery for children (WMTB-C; Pickering & Gathercole, 2001): Digit recall and Backward digit recall. Assessing digit recall, the participant is asked to repeat a sequence of digits read aloud by the experimenter, in the same order. After two practice items, sequences of three digits have to be repeated. When at least four of six presented sequences are repeated correctly, the reading specialist proceeded to the test items with one more digit, until less than four sequences were repeated correctly. Assessing Backward digit recall, the participant is asked to repeat a sequence of digits read aloud by the reading specialist in reverse order. The procedure of test administration resembles that of digit recall. Results were evaluated in terms of raw scores of the total number of correct responses.

**Block recall**

This input variable was assed using a subtest of the Working memory test battery for children (WMTB-C; Pickering & Gathercole, 2001). The participant was asked to imitate the sequence of blocks positioned on a board pointed out by the experimenter. The procedure of test administration resembled that of digit recall. Results were evaluated in terms of raw scores of the total number of correct responses.

**Word recall**

These input variables were assessed using the 15-Words test for children [15-Woordentest voor kinderen, gordijn-versie] (Kingma & Van den Burg, 2003). The participant was asked to listen to a list of fifteen verbally presented words on an audio-cd played by the experimenter, and repeat as many words as possible once the list was completed, in random order. This procedure was repeated five times, each time the participant was asked to name all recalled words, including words that were already named in previous rounds. After 30 minutes (in which nonverbal tasks were presented that did minimally address memory), the participant was asked to recall as many words as possible in random order once more. This assessment resulted in two measures: 1) Reproduction score: The sum of correctly named words during the first five rounds, and 2) Recall score: The number of words correctly recalled after the 30-minute pause.

**Word decoding**

This assessment consisted of the One-Minute Test [Eén Minuut-Test, EMT] (Brus & Voeten, 1972). The test consisted of words listed on a sheet, in ascending order of difficulty. The words had to be read aloud as fast as possible. The raw score was the number of words read accurately within one minute. To compare word decoding level scores on various assessment moments, norm tables in the manual were used to transfer raw scores in standardized c scores.

**Study 3**

**Rhyme**

Participants were requested to respond with a rhyme on 30 auditory presented words. The measure consisted of the number of correct responses; both existent and nonexistent words were accepted.

**Rhyme prime**

Participants were requested to complete the last word of 30 auditory presented sentences. Prior to each sentence a rhyming prime was presented. The measure consisted of the number of correct responses.

**Auditory synthesis**

Participants were requested to merge auditory presented word parts (e.g., syllables, starting phoneme and rest, phonemes) to 24 existing words. The measure consisted of the number of correctly synthesized words.

**Phoneme deletion**

Participants were auditory presented with 30 words and each time were requested to pronounce the remaining word when omitting a specific part, syllable or phoneme. The measure consisted of the number of correctly pronounced words.

**Letter naming**

Participants were visually presented with 20 letters and were asked to name them. The measure consisted of the number of correctly named letters.

**Word decoding**

This assessment consisted of the One-Minute Test [Eén Minuut-Test, EMT] (Brus & Voeten, 1972). The test consisted of words listed on a sheet, in ascending order of difficulty. The words had to be read aloud as fast as possible. Test raw score was the number of words read accurately within one minute.

**References**

Blomert. L. & Vaessen, A. (2009). *3DM Dyslexie. Cognitieve analyse van lezen en spellen. Gebruikershandleiding* [3DM Dyslexia. Cognitive analysis of reading and spelling. User manual]. Boom.

Brus, B. Th., & Voeten, M.J.M. (1972). *Een Minuut Test* [One Minute Test]*.* Berkhout.

Kingma, A. & Van den Burg, W. (2003). Drie parallelversies van de 15-woordentest voor kinderen: Handleiding en normering [Three parallel versions of the 15-word test for children: Manual and standards]. Afdeling Kinderoncologie, Universitair Medisch Centrum Groningen.

Pickering, S., & Gathercole, S. (2001). *Working Memory Test Battery for Children (WMTB-C).* The Psychological Corporation.

Schlichting, L. (2005). *Peabody Picture Vocabulary Test (PPVT-III-NL). Handleiding.* Harcourt.

Verhoeven, L., Keuning, J., Horsels, L., & van Boxtel, H. (2013). *Testinstrumentarium taalontwikkelingsstoornissen* [Test for language impairment]. Cito.

Wechsler, D., & Naglieri, J. A. (2008). *Wechsler Nonverbal Scale of Ability. Nederlandstalige Bewerking (WNV NL)*. Pearson.
